# Measuring clinical outcomes in adult ADHD clinics: psychometrics of a new scale, the adult ADHD Clinical Outcome Scale

**DOI:** 10.1192/bjo.2024.739

**Published:** 2024-10-14

**Authors:** Dimitrios Adamis, Jasmin Singh, Iulian Coada, Margo Wrigley, Blánaid Gavin, Fiona McNicholas

**Affiliations:** Sligo Mental Health Services Adult ADHD Clinic, Sligo, Ireland; Department of Psychiatry, University of Galway, Ireland; and Department of Psychiatry, University of Limerick, Ireland; Sligo Mental Health Services Adult ADHD Clinic, Sligo, Ireland; National Clinical Programme for Adult ADHD, Ireland; Department of Psychiatry, University College Dublin, Ireland; Department of Psychiatry, University College Dublin, Ireland; Lucena Clinic, Rathgar, Dublin, Ireland; and CHI Crumlin, Dublin, Ireland

**Keywords:** Attention-deficit hyperactivity disorder, adults, ADHD Clinical Outcome Scale, routine clinical outcome monitoring, psychometrics

## Abstract

**Background:**

Adult attention-deficit hyperactivity disorder (ADHD) clinics are in their infancy in Ireland and internationally. There is an urgent need for clinical evaluation of these services. Until now, clinical outcomes have relied mainly on functional scales and/or quality of life. However, adult ADHD is a longstanding disorder with many comorbidities. Although medication for ADHD symptoms can have immediate effects, co-occurring problems may take considerably longer to remediate.

**Aims:**

To present the psychometrics of a short outcome measure of key clinical areas including symptoms.

**Method:**

The ADHD Clinical Outcome Scale (ACOS), developed by the authors, is a clinician-rated scale and was administered in consecutive adults attending an ADHD clinic. A modified version was completed by the participant. A second clinician independently administered the scale in a subsample. ACOS consists of 15 items rated on a Likert scale. Two self-report scales, the Adult ADHD Quality of Life Questionnaire (AAQoL) and Weiss Functional Impairment Rating Scale (WFIRS), were also administered.

**Results:**

The mean age of 148 participants was 30.1 years (s.d. = 9.71), and 81 were female (54.7%). The correlation for interrater reliability was *r* = 0.868, and that between the participant and clinician versions was *r* = 0.663. The intraclass correlation coefficient for the internal consistency was 0.829, and the correlations for concurrent validity with total AAQoL and WFIRS scores were *r* = −0.573 and *r* = 0.477, respectively. Factor analysis revealed four factors: (a) attentional/organisational problems; (b) hyperactivity/impulsivity; (c) comorbidities; and (d) alcohol/drug use, self-harm and tension in relationships.

**Conclusions:**

The psychometrics of the ACOS are promising, and the inclusion of typically co-occurring clinical domains makes it suitable for use as a clinician-rated outcome measure in every contact with patients attending adult ADHD clinics.

Attention-deficit hyperactivity disorder (ADHD) is a neurodevelopmental disorder characterised by significant symptoms of either inattention or hyperactivity and impulsiveness or a combination of the two. Although it has been seen as a disorder of childhood, there is now evidence from longitudinal and epidemiological studies that it persists into adult life or may be first diagnosed during adulthood.^1,2^ Symptoms of hyperactivity and/or impulsivity may decline with increasing age, but inattentiveness may continue. Similarly, symptoms of mood dysregulation, sleep disturbances, procrastination and low frustration tolerance tend to persist or worsen throughout adult life.^3,4^ These observations and studies have resulted in increased interest in the clinical presentation of ADHD in the adult population at the same time as specialist adult ADHD clinics have started to be developed internationally and in Ireland. For the evaluation of clinical outcomes, most of those clinics use ADHD symptom scales (to rate the improvement or persistence of ADHD symptoms) such as the Adult ADHD Self-Report Scale, functional scales such as the WEISS Functional Impairment Rating Scale (WFIRS), measures of quality of life such as the Adult ADHD Quality of Life (AAQoL) scale, clinical global impression scales such as the Clinical Global Impression-ADHD-Severity scale, or a combination of these. Under the model of care for adult ADHD in Ireland, use of the WFIRS for monitoring of treatment is recommended. However, although all of these scales have been well validated and most of them are applicable to adult ADHD, they are limited in their ability to measure clinical outcomes for the following reasons: (a) they investigate and measure only specific domains of ADHD (e.g. function or quality of life, or only symptoms of ADHD); (b) they are lengthy (e.g. the WFIRS consists of 69 items, the AAQoL of 29 and the World Health Organization Quality of Life-BREF of 26); and (c) they ignore commonly occurring symptoms of comorbid disorders in adults with ADHD, which is the rule rather than the exception. In addition, they may take a long time to reflect change, as they measure functional and behaviour patterns well established throughout childhood and adulthood, whereas medication for ADHD can remediate symptoms quickly – if they work for the individual – and, consequently, can change behaviours and patterns in the longer term.^5^ Therefore, a brief scale which addresses the main symptoms of ADHD and common comorbidities, together with functionality problems, would be helpful for routine measurement of clinical outcomes.

Furthermore, clinical measurement outcome scales are particularly useful in determining the effectiveness of interventions and planning high-quality services and treatment strategies.^6^ Consequently, the implementation and use of an outcome scale in clinical practice could provide insights regarding treatment outcomes, service quality and more constructive ways of developing services for adults with ADHD according to their needs.^7^ Moreover, collection, analysis and feedback to clinicians of observational data can enhance evidence-based interventions.^8,9^ Generally, a clinical outcome scale is beneficial in aiding clinicians to track patient progress, adding effectiveness data to supplement efficacy data, informing clinical governance and policy, and, ultimately, improving outcomes for patients.^10^

Ideally, a clinical outcome measurement scale needs to be: quick and simple to use; applicable to a wide range of symptoms, including functioning; acceptable to clinicians; sensitive to change; and, of course, reliable.^7,11^

In the present work, we have developed such an instrument. Here, we provide its psychometrics, which require replication in further studies and in different settings.

## Method

### Development of ACOS

The scale was based on a review of the relevant literature, together with previous research in an adult mental health services setting.^1,12^ In addition to rating core symptoms of ADHD, functionality in school/college/work and everyday life has been included, as have common comorbidities such as anxiety, depression, sleep disorders and addictions. Although the scale was designed for clinical use, a self-report version has also been developed for participants. Exploratory work included content validation by asking ten experts to rate a pool of 20 items as ‘essential’, ‘useful, but not necessary’ or ‘not necessary’.^13^ Then, other peers were asked to evaluate feasibility and language issues, followed by piloting with patients for feedback and to test comprehension. The final scale includes 15 items (for the full scale, see Supplementary material available at https://doi.org/10.1192/bjo.2024.739) as follows: hyperactivity/restlessness, attention difficulties, temper/anger outbursts, problems with addictions, emotional dysregulation, disorganisation, impulsivity, tension in relationships, self-harm, procrastination, anxiety, depression, sleep, college/work difficulties and difficulties in everyday life. Each item is rated on a five-point Likert scale from 0 = no problem to 5 = very severe problem. The ratings refer to the problems which participants had experienced in the 2 weeks before assessment. Completion of the questionnaire by the clinician typically took 3 min after the clinical interview.

### Design of the study

This was an observational, pragmatic study investigating the psychometrics of a new scale.

### Setting/participants

Consecutive patients who had been referred to a tertiary specialist clinic for adult ADHD (18 years and above) were approached for recruitment to this project. In the Republic of Ireland, adult ADHD clinics were developed under the National Clinical Programme and accept referrals from adult community mental health teams; they work in an out-patient setting only. Most of the referrals involve patients with moderate to severe symptoms of ADHD and often with comorbidities. Participants referred to ADHD clinics that did not fulfil the criteria for an ADHD diagnosis were excluded from the analysis (*n* = 16).

### Measurements/scales

- Demographics (age, gender) and ADHD-related medication (from files).
- The ACOS (as above).
- The AAQoL, which was developed and validated by Brod and colleagues to measure quality of life in patients with ADHD; the psychometrics of the scale have been investigated in various studies, and it is considered to be a valid and reliable instrument.^14–16^ It consists of 29 items, rated by patients on a five-point Likert scale ranging from 1 to 5. It yields a total score (based on all items) and four subscale scores: life productivity, psychological health, life outlook and relationships. After reversing scores and transforming them to a scale from 0 to 100, higher scores indicate better quality of life.
- The WFIRS^17^ is a self-report scale consisting of 69 items which cover seven domains of functioning. Each item is rated on a four-point Likert rating scale from zero (never or not at all) to three (very often/very much). Mean scores can be calculated by omitting items with a missing or ‘not applicable’ response. A higher total mean score (or on each domain) indicates greater functional impairment. The WFIRS is recognised to have good psychometric properties and is widely used in research and clinical practice.^18,19^

### Procedure

Consecutive patients referred to adult ADHD clinics who fulfilled the criteria for ADHD according to the DSM-5 were rated with the ACOS after the clinical interview by one clinician. At the end of the clinical assessments, participants also self-rated using the ACOS (patient version), the AAQoL and the WFIRS. In a subsample (*n* = 60), a second clinician briefly interviewed the participants again and carried out a second rating of ACOS blind to the findings of the first clinician for the purpose of investigating interrater reliability. Approximately 3 months later during follow-up, a sample (*n* = 83) of the initial participants was re-examined, and the ACOS was administered again (both clinician and patient versions, without access to previous ratings) to investigate the capability of ACOS to detect clinical changes over time.

### Ethics statement

This study received ethical approval from Sligo University Hospital (no. 912; 5 April 2022:). Informed consent was obtained in writing from participants.

### Statistical analysis

All data were coded and entered into IBM SPSS v. 24 for Windows. Descriptive statistics (mean, median, s.d. and frequencies) were calculated as appropriate for all questionnaires. The interrater reliability was established by comparing the ACOS scores obtained by the two raters. To estimate the intraclass correlation coefficient, a two-way random effects model was used, assuming that both population effects and measure effects were random (type C, one intraclass correlation coefficient): MS_R_ − MS_E_/MS_R_ + (*k* − 1)MS_E_, where MS_R_ is the mean square for rows, MS_E_ is the mean square error and *k* is the number of items. Evidence of concurrent validity was demonstrated by comparing ACOS scores with the totals of AAQoL and WFIRS scores and the scores on the psychological domain of the AAQoL (which is considered to be closest to clinical symptomatology) using Pearson correlations. The ability of the ACOS to detect clinical changes over time was investigated by comparing initial ACOS scores with the scores provided after a 3-month period (follow-up) using paired *t*-tests. Similarly, paired *t*-tests were used to measure discrepancies between clinician ratings and patient ratings for each item of ACOS. Given that the comparisons were paired and expected (planned comparisons), no corrections for multiple comparison were used. Finally, to uncover the underlying structure of the ACOS, an exploratory factor analysis was performed, using principal component analysis extraction with promax rotation (as the factors were expected to be correlated). The number of factors was determined by using Kaiser's rule, which requires eigenvalues of greater than 1, and visually using a scree plot.

## Results

### Descriptive statistics

The sample consisted of 148 adult participants diagnosed with ADHD. Eighty-one were females (*n* = 81, 54.7%), and the median age was 28 with an interquartile range of 17 (mean = 30.1, s.d. = 9.71). The mean total ACOS score for the clinician version was 39.42 (s.d. = 10.33), and that for the patient version was 38.99 (s.d. = 12.1). The mean total AAQoL score was 36.93 (s.d. = 14.27), and the mean score on the psychological health domain was 35.76 (s.d. = 19.93). Finally, the total mean score of the WFIRS was 1.24 (s.d. = 0.47). Of the 148 participants, four (*n* = 4, 2.7%) were already on ADHD medication.

### Concurrent validity

The concurrent validity was examined by comparing the ACOS clinician scores with total AAQoL scores, AAQoL psychological health domain scores and total WFIRS scores using Pearson correlations. The total correlation between ACOS and total AAQoL scores was *r* = −0.573 (*P* < 0.001), that between ACOS and AAQoL psychological health scores was *r* = −0.547 (*P* < 0.001), and that between ACOS and WFIRS scores was *r* = 0.477 (*P* < 0.001).

### Interrater reliability

The Pearson correlation between the two raters (*n* = 60) for the total ACOS score was *r* = 0.868 (*P* < 0.001), and the agreement (Cronbach's alpha) was 0.928.

### Intraclass correlation coefficient

The intraclass correlation (two-way random effects model) was found to be 0.829 (*F* = 5.843, d.f._1_ = 146, d.f._2_ = 2044, CI 95%: 0.786–0.867, *P* < 0.001).

### Clinical change

A subsample (*n* = 83) were invited back for a 3-month follow-up assessment. Thirty-six of them (43.7%) were not on medication (27 because it had not been prescribed for various reasons, and nine because they had stopped taking it owing to side-effects). There was no significant difference in basic demographics (age and gender) between those with follow-up and those without (*n* = 83 *v*. *n* = 65). For age, Mann–Whitney *U* = 2683.5, *z* = −0.054, *P* = 0.957; for gender, *χ*^2^ = 0.799, d.f. = 1, *P* = 0.371. At follow-up, the same clinical rater repeated the ACOS, whereas the participant completed the ACOS patient version and two other self-report scales (AAQoL and WFIRS). Paired *t*-test between the first and second ACOS scores showed significant differences (*t* = −4.745, d.f. = 82, *P* < 0.001). Similarly, differences were found for the patient version (paired *t*-test, *t* = −4.615, d.f. = 80, *P* < 0.001) as well as for the AAQoL and WFIRS (paired *t*-tests; *t* = 5.335, d.f. = 79, *P* < 0.001 and *t* = 4.017, d.f. = 78, *P* < 0.001, respectively).

### Correlation between clinician and patient versions of the ACOS

There was a strong and significant correlation between the two versions of the ACOS (*r* = 0.663, *P* < 0.001). Discrepancies between the ratings were further examined by using paired *t*-tests for each of the 15 items (some items had missing values). The results are shown in Table 1.

**Table 1:** Paired *t*-test for each item between clinician and patient versions of the ADHD Clinical Outcome Scale (ACOS)

		Paired differences					*t*	d.f.	Sig.
		Mean	s.d.	s.e.	95% CI				
					Lower	Upper			
Pair 1	ACOS-C Q1, ACOS-P Q1 (hyperactivity)	−0.297	1.103	0.091	−0.477	−0.118	−3.278	147	**0**.**001**^a^
Pair 2	ACOS-CQ2, ACOS-P Q2 (attention)	0.027	1.176	0.097	−0.164	0.219	0.281	146	0.779
Pair 3	ACOS-CQ3, ACOS-P Q3 (temper/anger)	−0.122	1.234	0.101	−0.322	0.079	−1.199	147	0.232
Pair 4	ACOS-C Q4, ACOS-P Q4 (alcohol and drugs)	0.476	1.094	0.090	0.298	0.654	5.279	146	**<0**.**001**
Pair 5	ACOS-C Q5, ACOS-P Q5 (emotional dysregulation)	−0.358	1.119	0.092	−0.540	−0.176	−3.894	147	**<0**.**001**
Pair 6	ACOS-C Q6, ACOS-P Q6 (disorganisation)	−0.190	1.386	0.114	−0.416	0.036	−1.666	146	0.098
Pair 7	ACOS-C Q7, ACOS-P Q7 (impulsivity)	−0.493	1.431	0.118	−0.726	−0.261	−4.193	147	**<0**.**001**
Pair 8	ACOS-C Q8, ACOS-P Q8 (tension in relationships)	0.270	1.254	0.103	0.067	0.474	2.622	147	**0**.**010**
Pair 9	ACOS-C Q9, ACOS-P Q9 (self-harm)	0.405	0.961	0.079	0.249	0.561	5.134	147	**<0**.**001**
Pair 10	ACOS-C Q10, ACOS-P Q10 (procrastination)	−0.156	1.312	0.108	−0.370	0.057	−1.446	146	0.150
Pair 11	ACOS-C Q11, ACOS-P Q11 (anxiety)	0.061	1.241	0.102	−0.141	0.262	0.596	147	0.552
Pair 12	ACOS-C Q12, ACOS-P Q12 (depression)	0.388	1.190	0.098	0.194	0.582	3.949	146	**<0**.**001**
Pair 13	ACOS-C Q13, ACOS-P Q13 (sleep)	−0.223	1.211	0.100	−0.420	−0.026	−2.240	147	**0**.**027**
Pair 14	ACOS-C Q14, ACOS-P Q14 (college/work difficulties)	0.299	1.333	0.111	0.079	0.518	2.688	143	**0**.**008**
Pair 15	ACOS-C Q15, ACOS-P Q15 (difficulties in personal life)	0.189	1.220	0.100	−0.009	0.387	1.887	147	0.061

ADHD, attention-deficit hyperactivity disorder; Sig., significance.

a. Bold indicates significant differences. A negative value of the mean indicates that patients rated the item higher compared with clinicians.

Patient ratings were significantly higher than clinician ratings for hyperactivity, emotional dysregulation, impulsivity, and sleeping problems, whereas they were significantly lower for problems with alcohol and drugs, tension in relationships, self-harm, depression and difficulties in work/college (Table 1).

### Exploratory factor analysis

An exploratory factor analysis was performed using principal component analysis extraction with promax rotation and Kaiser normalisation. The results, together with the scree plot, indicated a four-factor solution that explained 61.1% of the variance. The Kaiser–Meyer–Olkin measure of sampling adequacy was 0.803, and Bartlett's test of sphericity gave *P* < 0.001 (*χ*^2^ = 719.49, d.f. = 105), both indicating that the data were suitable for factor analysis (adequate sampling, multivariate normal distribution and equal variance). The four factors and the loadings are presented in Table 2. In Table 3 the communalities and the total variance explained are presented.

**Table 2:** Exploratory factor analysis of the ADHD Clinical Outcome Scale (ACOS); loadings of the four-factor solution

	Factors			
	Attention/function	Dysregulation	Comorbidities	Risk-taking behaviours
ACOS Q6 Disorganisation	**0.859**			
ACOS Q10 Procrastination	**0**.**813**	0.134		−0.229
ACOS Q14 College/work difficulties	**0**.**756**	−0.242		0.251
ACOS Q2 Attention problems	**0**.**674**		0.123	
ACOS Q15 Difficulties in everyday personal life	**0**.**616**		0.191	0.153
ACOS Q7 Impulsivity		**0**.**862**	−0.213	
ACOS Q1 Hyperactivity /restlessness		**0**.**824**		−0.139
ACOS Q5 Emotional fluctuation (dysregulation)		**0**.**644**		0.331
ACOS Q3 Temper/anger outburst		**0**.**640**	0.318	
ACOS Q12 Depression problems		−0.139	**0**.**794**	−0.172
ACOS Q11 Anxiety problems		0.180	**0**.**777**	0.192
ACOS Q13 Sleep problems	0.276	−0.147	**0**.**391**	
ACOS Q9 Self-harm	−0.103	0.127	0.208	**0**.**757**
ACOS Q4 Problems with alcohol and drugs	0.151	0.397	−0.404	**0**.**676**
ACOS Q8 Tension in relationships		−0.139	0.166	**0**.**509**

ADHD, attention-deficit hyperactivity disorder.

Bold indicates the higher loadings in each factor.

**Table 3:** Exploratory factor analysis of the ADHD Clinical Outcome Scale (ACOS); communalities and variance explained

Communalities						
				Initial		Extraction
ACOS Q1 Hyperactivity/restlessness				1.000		0.615
ACOS Q2 Attention problems				1.000		0.507
ACOS Q3 Temper/anger outburst				1.000		0.579
ACOS Q4 Problems with alcohol and drugs				1.000		0.533
ACOS Q5 Emotional fluctuation (dysregulation)				1.000		0.626
ACOS Q6 Disorganisation				1.000		0.714
ACOS Q7 Impulsivity				1.000		0.727
ACOS Q8 Tension in relationships				1.000		0.602
ACOS Q9 Self-harm				1.000		0.629
ACOS Q10 Procrastination				1.000		0.712
ACOS Q11 Anxiety problems				1.000		0.630
ACOS Q12 Depression problems				1.000		0.691
ACOS Q13 Sleep problems				1.000		0.391
ACOS Q14 College/work difficulties				1.000		0.593
ACOS Q15 Difficulties in everyday personal life				1.000		0.607
Total variance explained						
Factor	Initial eigenvalues			Extraction sums of squared loadings		
	Total	Percentage of variance	Cumulative percentage	Total	Percentage of variance	Cumulative percentage
Attention/function	4.711	31.406	31.406	4.711	31.406	31.406
Dysregulation	1.872	12.478	43.884	1.872	12.478	43.884
Comorbidities	1.437	9.578	53.462	1.437	9.578	53.462
Risk-taking behaviours	1.138	7.587	61.049	1.138	7.587	61.049

ADHD, attention-deficit hyperactivity disorder.

The underlying structure of the ACOS involves four main factors: (a) attention problems together with functional and occupational problems, (b) impulsivity and hyperactivity together with emotional dysregulation and temper/anger outbursts, (c) observed comorbidities with ADHD (depression, anxiety and sleep disturbances) and (d) comorbidities with risk-taking behaviours (self-harm, alcohol and drug use, and tension in relationships) (Table 2).

## Discussion

The results demonstrate that the ACOS is a valid scale with significant correlation with other scales (AAQoL and WFIRS) commonly used for measuring outcomes in clinical trials, research and some adult ADHD clinics. Significant but moderate correlations were found between ACOS and WFIRS scores (*r* = 0.477; *P* < 0.001) and between ACOS and both total AAQoL scores (*r* = −0.573; *P* < 0.001) and psychological health domain scores (*r* = −0.547; *P* < 0.001). The imperfect correlations may have been because the WFIRS and AAQoL measure different constructs (functionality and quality of life, respectively), whereas the ACOS measures symptoms, comorbidities and risk-taking behaviours, in addition to functionality. The AAQoL and WFIRS were created to address the impact of ADHD symptoms on the lives of patients, and some of their items overlap with questions targeting ADHD symptoms or impairment caused by ADHD.^14^ Another possible reason is that the ACOS is clinician-rated, whereas the AAQoL and WFIRS are self-rated. There are differences between clinician-rated and self-rated scales, as well as between informants; however, there is value in taking a multisource approach to assessments.^20,21^ In any case these three scales are not antagonistic but complementary to each other, although perfect correlation is impossible. The correlations between the ACOS and comparator scale AAQoL were negative because a higher score on the AAQoL indicates better quality of life, whereas a higher score on the ACOS indicates a greater severity of problems from ADHD. The opposite was found for the WFIRS, corresponding to higher scores indicating poorer functioning.

Furthermore, the interrater reliability and agreement between the two clinicians was high. This was owing to the simplicity of the scale and the clinically meaningful instructions provided for rating the items. Clinicians do not need any previous training to use the scale. The ACOS also demonstrated excellent internal consistency with an intraclass correlation coefficient value of >0.75.^22^

In addition, the ACOS was found to be as sensitive to change as the two comparator scales. It is worth noting that nearly all the participants at the first assessment had been diagnosed with ADHD for the first time in adulthood and were not on medication for ADHD. By the second assessment, treatment (mainly medication) had been initiated; this perhaps explains the substantial changes in the short timeframe. Changes in clinical outcomes are also dependent on the severity of symptoms, the setting and additional diagnoses.^10^ To adjust for confounding effects, we calculated the corresponding changes on the AAQoL and WFIRS and found that the change for the ACOS was comparable in direction and degree with those of the two other scales.

The correlation between the clinician and patient versions of the ACOS was good, although the ACOS was designed for clinician use after clinical review This correlation shows that the severity of problems and/or symptoms as objectively noticed by the clinician was very close to what the patient endorsed. However, there were significant discrepancies for some of the items. Patients underrated alcohol and drug problems, tension in relationships, self-harm, depression and difficulties in work/college compared with the clinician. Underestimation of alcohol and drug use has been well documented.^23,24^. Similarly, discrepancies between self-reported scales and clinician ratings have been observed in other mental disorders including depression, suicidality, post-traumatic stress disorder and obsessive–compulsive disorder, where there is overrating or underrating of particular symptoms or of improvements in some of the symptoms. For instance, a meta-analysis of treatment studies of depression reported that effect sizes of treatment as assessed by self-administered scales were smaller than the effect sizes as assessed by clinician-rated measures.^25^ Self-report measures are also typically influenced by social and cultural norms, stigma and settings. For instance, patients who are being assessed for ADHD may focus more on ADHD-related issues such as hyperactivity, impulsivity and emotional dysregulation, which are more important to them (and they scored higher on those items compared with clinician ratings) than depression or work difficulties. Moreover, they may perceive that they might be criticised if they emphasise self-harm, tension in relationships, alcohol/drug misuse or work difficulties, causing them to underate.^23,26^ Other possible explanations for these discrepancies between the self-reported scale and clinician-rated scale include: (a) that patients with ADHD often have deficits in executive function,^27^ which may affect self-report rating; and (b) owing to the presence of comorbidities, the widespread range of psychopathology may introduce measurement variance that complicates differences between self-report and clinician-rated scores. We could not determine the reasons for those discrepancies in the present work; there are many factors^28^ that can contribute to those discrepancies and need to be considered and controlled for, but this was outside the scope of this study. However, the magnitude of change was similar between self-report scales (the patient version of the ACOS, AAQoL and WFIRS) and the clinician-rated ACOS. Similar discrepancies in ratings of symptoms have been reported among self- and clinician-rated scales in depression; however, similar to our findings, the level of change was similar between patient and clinician ratings.^29^

Finally, the exploratory factor analysis revealed four underlying factors. The first factor included attention problems, together with functional and occupational problems (disorganisation, procrastination, college/work difficulties and difficulties in everyday personal life). The finding that attention problems, a main symptom of ADHD, was loaded together with functionality and quality of everyday life was not surprising. Previous research has shown that inattentiveness is a strong mediator of ADHD-specific quality of life and functionality, compared with hyperactivity and/or impulsivity.^16,30,31^ In addition, disorganisation is mainly related to inattention; similarly, procrastination is related to inattention, and both are related to executive dysfunction.^32,33^

The second factor included impulsivity and hyperactivity together with emotional dysregulation and temper/anger outbursts. Emotional dysregulation has recently been investigated as a potential core symptom of ADHD. However, emotional dysregulation is more often observed in the combined ADHD or hyperactive/impulsive type than in the inattentive type of ADHD.^34,35^ Similarly, biological evidence (functional magnetic resonance imaging) suggests that hyperactivity/impulsivity is linked to reactive aggression and emotional dysregulation in patients with ADHD.^36^ Therefore, the ACOS seems to show good discrimination of these clusters of problems in adult ADHD.

The third factor included the most commonly observed comorbidities in adult ADHD (depression, anxiety and sleep disturbance), and the fourth factor included comorbidities with risk-taking behaviours (self-harm, alcohol/drug use and tension in relationships). The above comorbidities are the most common in adult ADHD,^12,37–40^ and the ACOS was specifically designed to rate these comorbidities. However, what appeared contrary to expectations was the loading of tension in relationships together with self-harm and substance misuse. Based on the extant literature, this would be expected to load in the second factor together with impulsivity/hyperactivity and anger. One possible explanation is the small sample size. In a larger sample, this item could load into the second factor, although the fact that the loading was quite high (0.55) argues against this. An alternative explanation, and one in need of further study, is a potential correlation of tension in relationships with drugs and alcohol use rather than attention or impulsivity.

### Limitations of the study

This study was conducted at only one centre, and the ACOS was developed in the same centre, increasing the probability of good psychometrics. Clinicians who rated the scales also worked together and were thus more likely to rate similarly, resulting in higher interrater reliability, however, they were blinded to each other's ratings. In addition, the sample involved consecutive patients and thus it was not truly random or representative of the total population. Also, the 3-month sample was a convenient sample in the sense that data for later or earlier assessments were not collected. Therefore, the psychometrics require retesting in different centres and/or clinics. Given that there are four different factors, overall total scores may not have meaning, but total factor scores need to be further investigated in future longitudinal studies.

In conclusion, the newly developed ACOS specifically designed for routine measurement of clinical outcomes in patients diagnosed with adult ADHD showed strong psychometric properties including concurrent validity, interrater reliability, intraclass correlation coefficient, sensitivity to clinical change, and high correlation between clinician and patient ratings, and the exploratory factor analysis demonstrated valid discrimination of four factors with high loading values. In the context of existing ADHD rating scales, the two most innovative characteristics of the ACOS appear to be that it is clinician-rated and that it is a broad measure of clinical outcomes (including functioning and other psychopathology in addition to ADHD symptoms). Therefore, we propose the use of the scale as a routine clinical outcome measure. However, it has only been tested in one clinic. The psychometrics of the scale need to be re-evaluated in other samples and settings of patients with adult ADHD.

## Supporting information

Adamis et al. supplementary material Adamis et al. supplementary material

## Data Availability

The data that support the findings of this study are available from the corresponding author, D.A., upon reasonable request.
