# Supplementary material for: Measuring clinical outcomes in adult ADHD clinics: psychometrics of a new scale, the adult ADHD Clinical Outcome Scale
Source: BJPsych Open. 2024 Oct 14;10(6):e180. doi: 10.1192/bjo.2024.739 (PMC11698188; doi:10.1192/bjo.2024.739)
Supplement: Adamis et al. supplementary material [file S2056472424007397sup001.docx]

**Supplementary material**

**ACOS** Clinician’s Scale

Name: dob

date

medications

Rating of items for the last 2 weeks

0 = no problem

1 = minor problem but no need to be addressed clinically.

2 = mild problem present but maybe not need clinical action

3 = moderately severe problem clinical action is needed

4 = severe problem

5= very severe problem

| **Problems/ symptoms/ behaviours** | **Score** |
| --- | --- |
| Hyperactivity /restlessness |  |
| Attention difficulties |  |
| Temper /anger outburst |  |
| Problems with alcohol and drugs |  |
| Emotional fluctuation (dysregulation) |  |
| Disorganisation |  |
| Impulsivity |  |
| Tension in relationships |  |
| Self-harm |  |
| Problems with procrastination |  |
| Anxiety problems |  |
| Depression problems |  |
| Sleep problems |  |
| College / work difficulties |  |
| Difficulties in the everyday personal life |  |
| **Total score** |  |

**ACOS** Patient’s scale (self-rating)

Name: dob

date

| **During the PAST 2 WEEKS, how troubled have you been by** | **Not at all** | **A little** | **Some**  **what** | **A lot** | **Very much** | **Extremely** |
| --- | --- | --- | --- | --- | --- | --- |
| Hyperactivity /restlessness | 0 | 1 | 2 | 3 | 4 | 5 |
| Pay attention when doing things | 0 | 1 | 2 | 3 | 4 | 5 |
| Temper /anger outburst | 0 | 1 | 2 | 3 | 4 | 5 |
| Problems with alcohol and drugs | 0 | 1 | 2 | 3 | 4 | 5 |
| Ups and downs in your mood | 0 | 1 | 2 | 3 | 4 | 5 |
| Organising things | 0 | 1 | 2 | 3 | 4 | 5 |
| Impulsivity | 0 | 1 | 2 | 3 | 4 | 5 |
| Tension in relationships | 0 | 1 | 2 | 3 | 4 | 5 |
| Self-harm | 0 | 1 | 2 | 3 | 4 | 5 |
| Postponing thigs | 0 | 1 | 2 | 3 | 4 | 5 |
| Anxiety problems | 0 | 1 | 2 | 3 | 4 | 5 |
| Depression problems | 0 | 1 | 2 | 3 | 4 | 5 |
| Sleep problems | 0 | 1 | 2 | 3 | 4 | 5 |
| College / work difficulties | 0 | 1 | 2 | 3 | 4 | 5 |
| Difficulties in the everyday personal life | 0 | 1 | 2 | 3 | 4 | 5 |
